# The Moderated Relationship of Appearance Valence on Appearance Self Consciousness: Development and Testing of New Measures of Appearance Schema Components

**DOI:** 10.1371/journal.pone.0050605

**Published:** 2012-11-30

**Authors:** Timothy P. Moss, Benjamin A. Rosser

**Affiliations:** 1 Centre for Appearance Research, Faculty of Health and Life Science, University of the West of England, Bristol, United Kingdom; 2 Clinical Psychology Training, College of Life and Environmental Sciences, University of Exeter, Exeter, United Kingdom; University of Bath, United Kingdom

## Abstract

This paper describes the creation and psychometric properties of two independent measures of aspects of appearance schematicity – appearance salience and valence, assessed by the CARSAL and CARVAL, and their relation to appearance self-consciousness. Five hundred and ninety two participants provided data in a web based task. The results demonstrate the sound psychometric properties of both scales. This was demonstrated by good item total characteristics, good internal reliability of each scale, and the independence of the two scales shown through principal components analysis. Furthermore, the scales show independent and moderated relationships with valid measures of appearance related psychosocial distress. Negatively valenced appearance information was associated with increased appearance self-consciousness. More crucially, the impact of negative valence on appearance self-consciousness was exacerbated by the moderating effect increased salience of appearance.

## Introduction

Research has established that appearance self-consciousness is not correlated with severity, size, or location of an objective difference of appearance [1]. These findings clearly demonstrate the importance of understanding the underlying psychological characteristics of individuals self-conscious of their appearance in order to better identify and support those who struggle with their appearance. We know that, counter-intuitively, levels of appearance self-consciousness are similar in the general population and visibly different populations [2]. Earlier work has hypothesised that the same basic psychological processes of making sense of one’s appearance are applicable across general and visibly different populations. While some of these processes have been well described (including, for example, social comparison processes, coping strategies, and use of social support [cf. 3]), within this paper we focus on issues related to the self-schema. Specifically, the purpose of this paper is to describe the development and evaluation of two brief measures of different aspects of the appearance self-schema and to demonstrate the way in which these two measures interact to better predict overall appearance self-consciousness. We conceptualise the appearance schema as the cognitive representation of organised information about the self in relation to appearance, which includes emotional and informational content about appearance, which serves also to guide information processing about one’s appearance.

Individual differences in appearance self-consciousness may be, at least in part, understood in terms of appearance self-schema [cf. 4, 5]. The appearance self-schema is the aspect of the self-concept which represents both the emotional evaluation of the self in relation to appearance (valence), as well as being the organising structure of that same information [6]. When negative appearance information is activated by external cues, there is a measurable increase in appearance distress [7], [8]. The impact of negatively valenced appearance information upon appearance self-consciousness is believed to be likely to be exacerbated by the salience of that information [9], although this has rarely been demonstrated in practice.

The self-schema is conceived of as a multi-faceted, dynamic and hierarchical information processing network, which guides behaviour through self-regulation, and guides information processing in relation to self-relevant information. The self has multiple sub-components, with varying levels of accessibility and perceived salience. Some self relevant information is perceived as more central– that is, more fundamentally similar to the overall way people perceive themselves [10], [11]. Greater centrality of this information increases the speed of processing material which is relevant to those aspects of the self. Importantly, central information is more *accessible*, and therefore more *salient*, and more often present in the working self-concept, than more peripheral information [11], and thus more involved in the interpretation and subsequent processing of social information. Salience can be related specifically to organisation of *appearance* information in the self-schema, and subsequently to the manifestation of appearance concern [8].

The importance of particular information with the self schema has implications for self-worth. That is, if valued (central) aspects of the self are seen as flawed, this will have a greater impact on overall level of esteem than if less important aspects are devalued [12], [13]. Work on the objectified self [14] has demonstrated the potential negative impact of internalising a negative social representation of the body and appearance. We argue here particularly that a combination of negatively evaluated appearance content and increased appearance salience is liable to be associated with greater levels of appearance related distress.

Cash and colleagues have investigated relations between appearance schematicity and appearance motivated behaviour [e.g., 15], culminating in the development of the 20 item ‘Appearance Schemas Inventory - Revised’ [16]. The measure of appearance schematicity is useful and widely used, but focuses on broader constructs than appearance salience as described above. Emotional processing in relation to appearance also features in the content of items within this scale – for example, in the item “*If somebody had a negative reaction to what I look like, it wouldn’t bother me*”. Furthermore, other items assess appearance contingencies (for example, “*My appearance is responsible for much of what’s happened to me in my life*.”). From this perspective, salience includes the extent to which appearance is a centrally defining feature of the self, and the level of dysfunctional investment in appearance. This makes the ASI-R invaluable in clinical investigation and many research settings, but it does not give us a focussed measure of the more specific construct under discussion here, the extent to which appearance information forms part of the working self-concept. Elsewhere, complex assessment procedures investigating salience of appearance information within the self-concept have been based on analysis of multiple, idiographic adjective checklists completed by participants. While this approach has had some success, it has also placed significant demand of participants and is impractical for routine use [8].

Further research and development of our understanding of appearance self-consciousness may benefit from differentiation of organisational (salience) and evaluative (valence) components of the appearance self schema, as well as considering the interrelation of these features. Moreover, brief assessment measures of these components may provide more pragmatic tools for researchers investigating appearance concern and/or appearance schematicity.

It is worth making explicit a subtle differentiation between “appearance self-consciousness” and the overlapping concept of “body dissatisfaction,” a component of body image. Body dissatisfaction is a broader concept than appearance self-consciousness, in that it includes unhappiness related to non-visible aspects of body. Grogan [17, p.4] defines it as “a person’s negative thoughts and feelings about his or her body”. Appearance self-consciousness is more specific, focussing upon negative feelings around the *appearance* of the body. For those most familiar with the tradition of weight related body image, (the majority of the body image work) this distinction may appear trivial. However, when working in areas of visible differences following disease and trauma, the distinction between self-consciousness of appearance, due to the visible manifestation of the body, and body image distress and dissatisfaction, based on other perceived attributes of the body (e.g., subjectively rated body instrumentality, functioning, vigour or health) is fundamental. In creating scales which, (unlike most other measures in the body image field), clearly isolates appearance from other aspects of body image, we aim to lay the path for clarity in measurement and consequent theorising in the future.

The aims of this research were therefore twofold: first, to develop brief, valid, and reliable measures of appearance salience and valence, and second, to evaluate both the independent or moderated predictive contribution of these factors in relation to the psychological difficulties of appearance self-consciousness.

It was hypothesised that convergent criterion validity of the salience measure would be demonstrated by moderate correlations with ASI-R sub-scales. A strong correlation would indicate the salience measure was not assessing anything different to the ASI-R. Only moderate correlations were predicted, as this would best indicate that the salience measure was conceptually related without being identical (although of course, we acknowledge the impact of sample size, and assume minimum impact of external non-measured variables and other non-hypothesis related factors which may impact on the size of the correlation coefficient). Discriminant criterion validity would be demonstrated by low correlations between salience and appearance self-consciousness (DAS24), positive affect and negative affect (PANAS), on the basis that salience of appearance itself is unrelated to the emotional impact of that salient information. While this cannot be proven through rejection of a null hypothesis, failure to reject the null hypothesis with a well-powered analysis gives confidence in discriminant validity. It was further hypothesised that the convergent criterion validity of the valence measure would be demonstrated by positive correlations with appearance self-consciousness, (DAS24), self-evaluative salience (ASI-R SES), and negative affect (NA), and a negative correlation with positive affect (PA). Again keeping in mind the same scientific issues in relation to the meaning of failure to reject the null hypothesis, it was hypothesised that divergent discriminant validity of the valence measure would be demonstrated by a non-significant correlation with ASI-R motivational salience, on the basis that motivational salience describes the extent to which persons attend to their appearance and engage in appearance-management behaviours, which is independent of the extent to which appearance is positively or negatively evaluated.

## Methods

### Participants

A power calculation was computed based on *a priori* estimations for multiple regression with three predictors, and taking conservative approach (power set at .9; alpha at .01, anticipated effect size .15) was adopted given the novelty of the scales. The calculation identified a minimum requirement of 198 participants. Typically [e.g., 18] a “very good” sample size for principal components analysis is taken as approximately 500 participants.

Participants were recruited through research focussed websites. (e.g. http://www.onlinepsychresearch.co.uk/) The websites utilised were selected because of associations with university research, their assessment of all research admitted (screening of protocols, evidence of ethical approval and university affiliation were required), and their prominence within the online research community.

Five hundred ninety two participants were recruited. Participant nationality was identified using participant determined categories, and was predominantly American (31%) although British, Hispanic, Japanese, Korean, Chinese, Mexican, Italian, Turkish, Vietnamese and South African were also reported. Participant demographic characteristics are summarised in table 1.

**Table 1:** Participant demographic information.

Female	80.8%
Male	19.2%
Undisclosed sex	10.3%
Age: Mean (SD)	25.1 (8.54) years
Ethnicity: White	65.0%
Ethnicity: Black African	8.8%
Ethnicity: Other	4.4%
Ethnicity: Undisclosed	21.8%

For test-retest analysis, participants were university students and were awarded course credit for participation. Forty one participants aged between 20 and 29 years (mean = 21.2; SD = 1.82) took part. Of these, 82.9% were women. All described their ethnicity as white, other than one who described themselves as Indian.

### Measures

An item pool for the salience and valence measures was developed consistent with recommendations in the established literature [19]. Items were generated from the clinical experience of authors and relevant literature, based on a careful theoretical operationalisation of the constructs (see below). This item pool was then refined on the basis of peer feedback from other experts in the appearance research community to ensure initial content validity.

#### Centre for Appearance Research Salience scale

The core construct of salience was operationally defined as “the extent to which appearance and physical self is brought into conscious awareness.” The Centre for Appearance Research Salience Scale (CARSAL) item pool consisted of 10 items with Likert scale response categories ranging from 1 (strongly disagree) to 6 (strongly agree). Three items were reverse scored. Higher scores for each item indicated increased salience of appearance within the self-concept – that is, appearance being part of the working self-concept – than a lower score. Items for this scale were specifically cognitive rather than affective or behavioural content.

#### Centre for Appearance Research Valence scale

The core construct of valence was operationally defined as “The extent to which the respondent evaluates her/his appearance in a positive/negative way”. The Centre for Appearance Research Valence Scale (CARVAL) item pool consisted of 12 items with the same response options as the CARSAL. Seven of the candidate items were reverse scored. Higher item scores indicated a more negatively valenced evaluation of appearance. Items for this scale were specifically affective and cognitive rather than behavioural.

For both scales, items were required to be applicable to objectively visibly different and also other general population respondents.

#### Derriford Appearance Scale –24

The Derriford Appearance Scale 24 [20] assesses the distress and difficulties experienced in living with a problem of appearance and can be used with a clinical and non-clinical population. The DAS-24 is a 24 item scale widely used psychometrically and clinically valid and reliable scale that was created primarily to address problems of appearance adjustment in clinical and non-clinical populations. The DAS-24 has high internal consistency (Cronbach’s alpha) of .92 and has good test-retest reliability of .82.

#### The Appearance Schemas Inventory – Revised

The Appearance Schemas Inventory – Revised (ASI-R) [16] measures appearance schematicity. The ASI-R consists of twenty statements with Likert scale response options. The measure produces one composite score of attractiveness schematicity, and two sub-factor scores of ‘Self-Evaluative Salience’ (SES) and ‘Motivational Salience’ (MS). SES refers to how salient attractiveness is to the individual, whereas MS is an assessment of how salient attractiveness is in motivating behaviour in the individual. The authors report good internal validity scores for whole and sub-scales of Cronbach’s alpha ranging from .80 to .91.

#### The Positive and Negative Affect Schedule

The PANAS [21] is a widely used measure of mood, consisting of ten positive and ten negative words each rated on a Likert scale, thus assessing Positive Affect (PA) and Negative Affect (NA). The authors report that for the Positive Affect Scale, Cronbach’s alpha was .86 to .90; for the Negative Affect Scale, 84 to .87.

### Procedure

For the main study, participants were provided with full information about the study by way of an introductory screen, which made them aware of the study content and their right to withdraw at any time. A consent screen, including a statement requiring the participant to acknowledge they were of eighteen years or above, was presented subsequently. Participants gave their consent to participate in the study by clicking the appropriate onscreen box. Following collection of informed consent, the measures were presented in a counterbalanced order. Standardised online debriefing was included, and the principal investigator’s contact details made available for any subsequent concern.

In order to investigate the stability of the instruments, test-retest reliability was assessed. There is no objectively agreed rule to determine an appropriate interval between test and retest, with existent examples ranging between a week and several years. Given that the stability of levels of appearance salience and valence were not known with great certainty a priori, a moderate interval of one month was selected in which we expected minimal variation in the underlying constructs. Participants were recruited through internal advertisement within their university. Data were collected in a classroom setting using paper-and-pencil administration of the CARSAL and CARVAL scales at two time points, one month apart.

### Ethics Statement

The study was scrutinised and accepted by the University Research Ethics Committee of the lead author. Participants gave informed consent, were assured of the right to withdraw without penalty, and assured of anonymity in written information and consent forms.

## Results

### Psychometric Properties of the Salience and Valence Scales

#### Internal structure of the salience scale

The 10 items in the initial pool were subjected to an item-total analysis to facilitate elimination of items demonstrating poor item-total correlation (r <.5), and ensure that inclusion of reverse scored items made no meaningful difference to the scale structure. Five items were excluded from further analyses at this stage. The subsequent analyses were conducted on the data set including (N = 592) and excluding 121 participants who did not complete every questionnaire item (N = 471). Exclusion of incomplete responses did not substantially alter the results; consequently, results for the full data set are reported hereafter.

Item-total analysis for the final items in the salience scale demonstrated Pearson’s r correlations between 0.74 and 0.81. Cronbach’s alpha was .90 (see table 2).

**Table 2:** Item-total analysis for the CARSAL salience scale.

	Corrected Item-Total Correlation
For me my appearance is an important part of who I am	.724
I am often aware of the way that I look to other people	.752
In most situations, I find myself aware of the way my face and body look	.810
I often think about the impression that the appearance of my face and body make	.759
I am usually conscious of my appearance	.773

The items were also evaluated to determine whether they had a normal distribution, and exclude items with floor or ceiling effects. It was not necessary to exclude any items at this stage.

#### Internal structure of the valence scale

To examine the internal structure of the valence scale, an item-total analysis was conducted. Three items were positively skewed (indicating item floor effects) and were excluded. One item was removed in the CARVAL analysis of study one data following spontaneous feedback included on the handwritten participant information sheet collected simultaneously for study two, indicating poor face validity. All of the remainder items had item-total correlations of r>0.5. Item-total analysis for the final items in the valence scale demonstrated Pearson’s r correlations between 0.72 and 0.84. Cronbach’s alpha for the scale was .93 (see table 3).

**Table 3:** Item-total analysis for the CARVAL valence scale.

Item	Corrected Item-Total Correlation
I am satisfied with my physical appearance*	.802
I don't like the way I look	.737
The way I look makes me feel good about myself*	.775
The way I look makes me unattractive	.716
My body and face look pretty much the way I would like*	.760
I feel bad about my body and my appearance	.714
I like to way I look*	.844
My appearance makes me feel attractive*	.758

* Reverse scored items.

#### Confirmation of construct identities

Principal components analysis with oblimin rotation (delta set to zero) was conducted to ensure that the conceptually independent constructs were also statistically independent. A priori theoretical assumptions assumed independence, but a varimax rotation, with forced orthogonal solution, would test this less well than the oblimin rotation which allowed a potential oblique relationship should the hypothesis of independence be incorrect. Two factors were identified, accounting for 70% of the variance. Items developed to represent CARSAL were all present in factor one, with item loadings between .80 and .87. No demonstrable loading was found between these items and the second factor. The items developed to represent CARVAL were all present in the second factor, with items loading between .76 and .90. Once again, no demonstrable loading was found between these items and the other factor. The correlation between the factors was r = −0.02, further supporting the independence of the factors.

#### Criterion validity

Power was calculated for all of the measure tested herein. For all except the CARSAL/DAS24 relationship, power approached 1.0. The CARSAL demonstrated convergent criterion validity through linear correlations with ASI-R motivational salience (r = .59, p<0.005, df = 480) and self-evaluative salience (r = .56, p<0.005, df = 480). Discriminant criterion validity of CARSAL was demonstrated by small effect correlations with DAS24 (r = .11, p<0.01, df = 523, power = 0.81), PA (r = .21, p<0.005 df = 469), and NA (r = .11, p = .02, df = 469).

The CARVAL demonstrated convergent criterion validity through linear correlations with DAS24 (r = .72, p<0.005, df = 523), ASI-R self-evaluative salience (r = .455, p<0.005, df = 480), PA (r = −0.39, p<0.005, df 471) and NA (r = .38, p<0.005, df = 471). Divergent discriminant validity of CARVAL was indicated by a non-significant correlation with ASI-R motivational salience (r = .04, ns, df = 480).

#### Regression analysis

Multiple regression analysis was conducted with DAS24 as the dependent variable; CARSAL and CARVAL were entered as independent variables. The overall model significantly predicted DAS24 score (F (2, 520) = 204.28, p<.001) accounting for 54% of variability in the dependent variable; both predictors provided significant independent contributions, CARVAL (β = .72) and CARSAL (β = .10). No evidence of multicollinearity was found.

**Figure 1:**
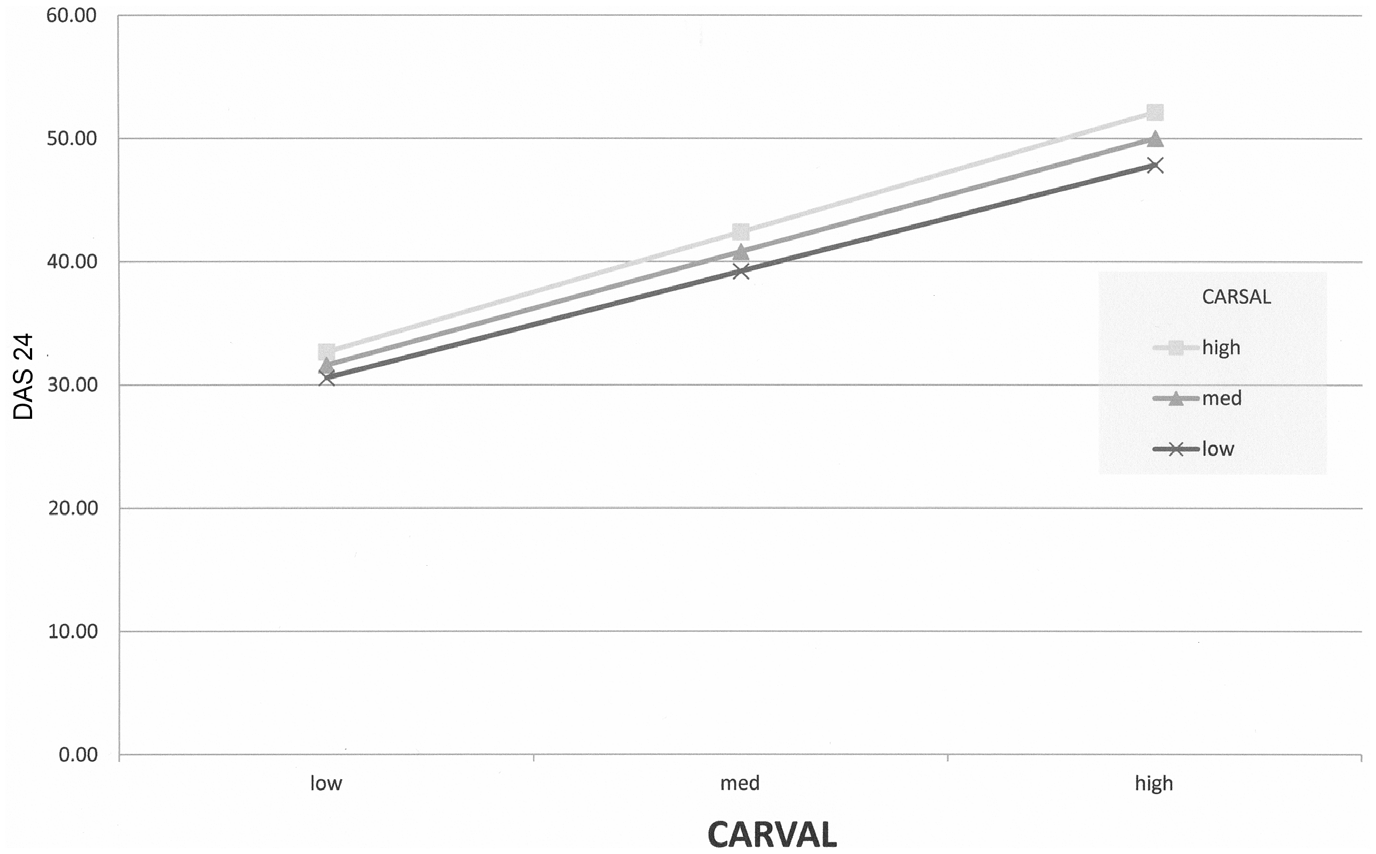
Moderation of CARVAL on DAS24 by CARSAL. Appearance self-consciousness and appearance valence relationship, based on continuous data split by low appearance salience (lower line), moderate appearance salience and high appearance salience (upper line). Graph produced on basis of continuous data by ModGraph-I [25].

It was predicted that, in addition to the independent effects of each scale described above, the relation between negative valence (CARVAL) and appearance adjustment (DAS24) would be moderated by the effect of salience (CARSAL). That is, in addition to the independent effects described above, poorer adjustment would also be predicted when the most negative valence was combined with the most salient appearance. To test this, an interaction (moderation) term was calculated by multiplication of the CARVAL and CARSAL scores, and entered into as a second step in the model (following entering CARSAL and CARVAL separately in step one). A small but significant adjusted R^2^ change was observed at this point (F(1, 519) = 7.02, p = 0.001) demonstrating that the moderation term was related to adjustment beyond main effects of salience and valence. As can be seen in figure 1, most appearance self-consciousness (high DAS24 scores) was associated with the most negative valence (high CARVAL), and this was exacerbated by higher levels of appearance salience (higher CARSAL).

#### Sex comparisons

ANOVA comparisons of CARSAL (F(1,529) = 6.7, p = .01, η_p_^2^ = 0.01) and CARVAL (F(1,529) = 14.8, p<.001, η_p_^2^ = 0.03) demonstrated significant sex differences respectively. In both instances, although female participants demonstrated higher mean responses; the difference between male and female mean response was within one standard deviation. Consistent with previous research [20], when compared with male participants, females demonstrated significantly higher levels of DAS24 assessed appearance concern (F(1,513) = 19.7, p<.001, η_p_^2^ = 0.04). Statistical power was in excess of 0.7 for each of these analyses, with alpha set at 0.05.

### Test – Retest Reliability

CARSAL and CARVAL measures demonstrated acceptable test-retest reliability calculated using Pearson’s r, of .74 and r = .89 respectively.

## Discussion

Current literature suggests that problems experienced by individuals troubled by their appearance are predominantly psychosocial [22] and can be associated with self-schema content and organisation. Prior to this study, measurement of appearance salience was complex and difficult [8]. The relationships between valence, salience, and appearance self-consciousness had been hypothesised but not clearly demonstrated. The objective of this work was to address these issues through the development and testing of appearance salience and valence measures, and examining their independent and combined role in the processing of appearance related information through their relationship with an established measure of psychosocial adjustment to appearance concern.

Item analysis of the CARSAL salience scale reduced 10 pool items to 5 items, which all strongly contributed to the measure and provided a good distribution of scores. Similarly, item analysis of the CARVAL valence scale demonstrated eight item measure selected from the initial pool of 12 items. Both measures were found to have a high level of internal consistency and robust item-total correlations, suggesting effective representation of the constructs of appearance salience and valence respectively. Furthermore, principal components analysis demonstrated the independence of the two constructs. The CARSAL demonstrated criterion validity through its association with motivational and self-evaluative salience, but weak relation with overall psychological adjustment to appearance, indicated through DAS24. This was consistent with hypotheses, and suggests that the measure was assessing salience, and furthermore, that appearance salience in isolation is not a predictor of appearance self-consciousness. Again, consistent with hypotheses, the CARVAL was clearly associated with appearance concern and emotional distress, as indicated through correlations with the ASI-R self evaluative salience, PANAS, and DAS24. Furthermore, it was shown through the low correlation between CARVAL and motivational salience that the extent to which persons attend to their appearance and engage in appearance-management behaviours need not be related to appearance self-consciousness; it is quite possible to feel positive about one’s appearance, and still invest time and effort in appearance management.

In addition to development of brief measures of appearance salience and valence, this research sought to clarify the independent and moderated relation of these constructs with appearance self-consciousness. Both salience and valence individually contributed to the regression model predicting appearance self-consciousness. The model accounted for over 50% of variability in concern, supporting claims that salience and valence are critical factors in understanding the experience and maintenance of appearance self-consciousness. Notably, valence contributed substantially greater variability compared with salience. Additional analyses revealed that the relation between valence and appearance self-consciousness is partially moderated by salience. This demonstrates that when negatively valenced appearance information is more salient – that is, more easily accessed and more frequently present in the working self-concept - individuals are at risk of increased appearance self-consciousness. A highly salient conception of appearance may serve as a vulnerability factor in the potential development of appearance concern, whereas negative valenced content relating to appearance is liable to determine the consequence of that vulnerability.

For some people, appearance features more centrally in their consciousness and is therefore more accessible to self-regulatory processes. In this way, the salient appearance schema is more likely to guide behaviour in relation to appearance (as indicated by the relationship between CARSAL and ASI-R MS) without necessarily being related to distress. However, the implications of these findings for information processing and behavioural and cognitive self regulation are at this stage hypothetical, and demand experimental analysis. Taken together, these results suggest that consideration must be given to both independent and interdependent relations between evaluative and organisational features of appearance within the self-concept.

There are limitations to this study. First, care must be taken in generalising to wider populations, particularly clinical populations, who were not actively recruited in this study. A cross-sectional design was used in the main part of this study. This methodology prohibits assessment of causality and does not account for potential dynamics in the nature of the variables considered. Work remains in mapping salience and valence to short and long term fluctuations in appearance self-consciousness across the lifespan. Differences between the mean scores of male and female participants were consistent with previous work on appearance self-consciousness. However, the extent to which underlying antecedent processes are similar or different across the sexes demands further investigation. Data were collected online which must be acknowledged as potentially influencing participant response: however, previous use of online versions of established measures does suggest comparability of responses with traditional paper methods [23]. Further work should be done to establish the user acceptability and face validity of the measures in clinical and non-clinical settings. Finally, the utility of these measures for use with clinical populations with more objectively identifiable visible differences is yet to be established. The extent to which these measures are acceptable, appropriate, and indicate similar underlying issues in relation to the self schema for participants with greater clinical need or more pronounced and distinctive appearance differences remains to be demonstrated.

The study findings contribute to our understanding of psychological experience of appearance concern. By demonstrating that adjustment involves a moderation of valence by the salience of appearance information, we have increased the scope for considering ways of working with the extremes of appearance self-consciousness through psychotherapy. However, we would sound a cautionary note. Although therapists may be alerted to two key foci in supporting their clients challenged by poor adjustment in relation to appearance (firstly, the importance of helping clients both re-evaluate their appearance in positive ways and secondly, to re-structure the organisation of the appearance self-schema to make appearance a less salient feature within the broader self-concept), to use our findings as the basis of this would be premature. We recognise that further, delicate work around the constructs of appearance salience and valence as used in a therapeutic context is required. Furthermore, practically, we recognise that these two aims may be therapeutically incongruent or difficult to implement; working with a client to make appearance more positive may work against the goal of making it less salient. For deeply embedded, core aspects of the self-schema which are resistant to change, it may therefore be more appropriate to work towards clients’ accepting these aspects of self and the associated emotional consequences [24].

### Conclusion

This research has demonstrated that the independent and interactional contribution of evaluative and organisational features of the self-concept may further our understanding of appearance self-consciousness. Furthermore, the outlined measures provide short-form assessment of appearance salience and valence, which we believe complement existing measures in the field of appearance psychology, providing practical tools for researchers investigating appearance schematicity specifically or individual conceptions of appearance more generally.
